# Metabolomics for organic food authentication: Results from a long-term field study in carrots

**DOI:** 10.1016/j.foodchem.2017.06.161

**Published:** 2018-01-15

**Authors:** Elena Cubero-Leon, Olivier De Rudder, Alain Maquet

**Affiliations:** European Commission, Joint Research Centre, Retieseweg 111, 2440 Geel, Belgium

**Keywords:** Carrots, Metabolomics, Authenticity, Conventional agriculture, Organic agriculture, Mass spectrometry, Chemometrics

## Abstract

Increasing demand for organic products and their premium prices make them an attractive target for fraudulent malpractices. In this study, a large-scale comparative metabolomics approach was applied to investigate the effect of the agronomic production system on the metabolite composition of carrots and to build statistical models for prediction purposes. Orthogonal projections to latent structures-discriminant analysis (OPLS-DA) was applied successfully to predict the origin of the agricultural system of the harvested carrots on the basis of features determined by liquid chromatography–mass spectrometry. When the training set used to build the OPLS-DA models contained samples representative of each harvest year, the models were able to classify unknown samples correctly (100% correct classification). If a harvest year was left out of the training sets and used for predictions, the correct classification rates achieved ranged from 76% to 100%. The results therefore highlight the potential of metabolomic fingerprinting for organic food authentication purposes.

## Highlights

• The agricultural origin of carrots could be predicted.
• Using yearly harvested samples allowed 100% correct classification of unknowns.
• Metabolomic fingerprinting showed potential for organic food authentication.

## 1 Introduction

Improved animal welfare, environmental protection and potential beneficial effects on human health are some of the reasons for the increasing demand for organic products. These products have a premium price over conventional products, which explain the appearance of fraud cases. Due to the lack of sound analytical methodology to distinguish organically and conventionally grown crops and in response to EU legislation (Regulation (EC) No 834/2007), analytical methods that can distinguish between both agricultural systems are needed.

Organic agricultural systems rely, among other characteristics, on the use of organic manures and biological pest controls and maintain the fertility of the soil by multiannual crop rotation including legumes and other green manure crops. In conventional agricultural systems plants are protected with chemical plant protection products. Therefore, organic production systems may increase environmental stress in plants; hence, resulting in accumulation of inducible protective secondary metabolites such as phenolic acids (Malik et al., 2009, Young et al., 2005, Zuchowski et al., 2008) and flavonoids (Mitchell et al., 2007).

Metabolomics allows the study of multiple metabolites in a cell, a tissue or an organism. Over the past decade, which saw new developments in analytical approaches such as mass spectrometry (MS) and chemometrics, the application of metabolomics to food authentication issues has gained increasing interest. To date, metabolomics based methods have not yet been taken up by the regulatory agencies for use in food authentication issues, although in some cases they have been proved to be efficient and shown clear benefits over traditional methods. The main advantage of the use of metabolomics in food authentication is its untargeted nature. Unexpected changes in the metabolite profile, i.e. by the addition of new adulterants, may be detected without the need of an *a priori* hypothesis which offers a great advantage ahead of the fraud performers (Cubero-Leon, Peñalver, & Maquet, 2014).

The application of fingerprinting for food authentication issues is still at an early stage and more studies with larger datasets are required to draw valid conclusions. Indeed, although some of these procedures have shown to be promising, more studies are needed that account for pedo-climatic factors, agricultural productions systems, genetics, processing, etc., to obtain models with wide applicability (for reviews see Cevallos-Cevallos et al., 2009, Cubero-Leon et al., 2014). For example, for the control of the European wine market, Commission Regulation (EC) No 555/2008 (European Commission, 2008) requires the EU Member States to collect every year grapes from selected growing areas, produce micro-vinified wines and subject them to isotopic analysis; the obtained data support the fight against fraud in the wine sector (mainly sugaring and watering).

Recently, metabolite fingerprinting applications have been used to study differences in organic *vs*. conventional production systems in wheat (Bonte et al., 2014, Kessler et al., 2015), maize (Röhlig & Engel, 2010), tomatoes and tomato-derived products and peppers (Novotná et al., 2012, Vallverdú-Queralt et al., 2011), grapefruits (Chen & Harnly, 2010), potatoes (Shepherd et al., 2014) and white cabbage (Mie et al., 2014). The results, however, are contradictory. In wheat small differences were found between the two production systems, which tended to disappear in mature grains whereas in tomatoes, maize, grapefruits and potatoes significant differences were found. Organic and conventional production systems in some cases could only be distinguished for a given year or a given cultivar due to the massive influence of these factors on the metabolite profiles (Bonte et al., 2014, Kessler et al., 2015). One of the main limitations of these studies is that they are based on relatively small samples sizes and/or other sources of variation such as cultivated varieties, geographical location or fertilisation practice are not considered. The lack of external validation sets using samples that are not part of the statistical models built for prediction is a major limitation in most of the studies published. The authenticity of the samples also needs to be assured. In this respect, samples from controlled experimental fields/studies should not be considered as authentic as they might not reflect the natural variation obtained from real agricultural practices.

The carrot (*Daucus carota* L.), a plant of the *Apiaceae* family, is one of the most economically important vegetables worldwide (FAO, 2016) and they can be consumed raw or in a wide variety of processed products. The objective of this study was to perform a comprehensive biochemical analysis based on untargeted liquid chromatography–mass spectrometry (LC–MS) metabolomics of carrots coming from different agronomic environments. The combined molecular features extracted were then used to build prediction models and classify the origin of new samples according to their agricultural practice using external validation sets. To the best of our knowledge, this is the first time that a metabolomics approach is used for organic food authentication purposes in a long-term (four years) field study and by using external validation sample sets to predict the origin of unknown samples.

## 2 Material and methods

The standards developed by the Metabolomics Standards Initiative (MSI; http://msi-workgroups.sourceforge.net/) were followed as much as possible.

### 2.1 Field samples

Carrot samples (*Daucus carota* L.) of Nerac and Namur varieties were obtained from the Walloon region of Belgium in four consecutive years (2005, 2006, 2007 and 2008). Carrots ‘Namur’ are an early cultivated variety (cultivar or cv.) suitable for spring harvesting whereas ‘Nerac’ carrots are a summer hybrid. Fields using conventional growth strategies were compared to fields using certified organic practices. The geographical location of all the fields sampled in this study is provided in Fig. S1 (Supplementary material). For information regarding dates of sowing and harvest, crop protection (pesticides), fertility management (fertilisers) and crop rotation details of the organic and conventional systems see Table S1 (Supplementary material).

In each location two paired fields were selected (maximum distance of 5 km), one with a conventional growing system and one with organic husbandry. Within each field, three parcels were selected (10 m × 10 m each), and the distance between parcels was 15 m. One sample was taken from every corner of each parcel. A fifth sample was taken in the centre of the parcel. From each field 15 carrots of similar size (biological replicates) were collected. Samples from each field were labelled and packed independently and sent the same day on ice to the laboratory. The fields were characterised by loamy soils. More information on the soil properties is detailed in Table S2 (Supplementary material). In every location carrots from both fields (organic and conventional) were harvested within less than two weeks except in 2005.

After delivery to the laboratory the carrots were kept at 4 °C for a maximum of 48 h. They were then washed and the top and bottom of the carrots (0.5 cm) removed, cut into slices and lyophilized during 72 h using a Christ Freeze dryer system/lyophilizer (Martin Christ GmbH, Osterode am Harz, Germany), after which they were homogenized with an ultracentrifugal grinder equipped with a 0.5 mm sieve (ZM 200, Retsch, Düsseldorf, Germany). The mean water content of the lyophilised carrots determined by Karl Fisher titration was 5.5%. Sample preparation was performed in a randomized way over 14 days. Samples were stored in sample bags at −80 °C freezer before analysis.

### 2.2 Reagents and chemicals

Water was purified using a Milli-Q Integral water purification system (Millipore, Bedford, MA, USA). Methanol (HPLC grade) used for the extraction was purchased from VWR (Leuven, Belgium), and the methanol used for chromatography (LC–MS grade) was supplied by Merck (Germany). Chloroform (purity ≥98%), formic acid (purity ∼98%), sodium formate (purity ≥99.9%) and isopropanol (≥99.9%) were purchased from Sigma-Aldrich (St. Louis, USA).

Vitamins: pyridoxine (C_8_H_11_NO_3_) and nicotinic acid (C_6_H_5_NO_2_); flavonoids: (−)-epigallocatechin (C_15_H_14_O_7_), (−)-epicatechin (C_15_H_14_O_6_), (+)-catechin (C_15_H_14_O_6_), rutin (C_27_H_30_O_16_); and phenolic acids: ferulic acid (C_10_H_10_O_4_), benzoic acid (C_7_H_6_O_2_), *trans*-cinnamic acid (C_9_H_8_O_2_), *p*-coumaric acid (C_9_H_8_O_3_), 4-hydroxybenzoic acid (C_7_H_6_O_3_) and chlorogenic acid (C_16_H_18_O_9_) were purchased from Sigma-Aldrich with purity ≥97% in all cases.

### 2.3 Sample extraction

Different standards (STDs) representing vitamins, flavonoids, and phenolic acids (see Section 2.2) already reported to be existent in carrots were chosen to assess the performance of the extraction method based on a procedure described by Ossipov et al. (2008).

A volume of 500 µL of ice-cold chloroform was added to 0.1 g of lyophilized carrot samples, followed by 300 µL of ice-cold methanol and 200 µL of ice-cold Milli-Q water. The samples were vortexed for five seconds and mixed in a Thermomixer Comfort (Eppendorf, Hamburg, Germany) at 4 °C at maximum speed (1400 rpm) for 1 h. Then, 0.5 mL of 20% aqueous methanol was added to the extract and the resulting biphasic system was vortexed for 5 s and mixed in the Thermomixer at 4 °C for 30 min at 1250 rpm. To separate the methanol/water and chloroform fractions the samples were centrifuged for 15 min at 10,000*g* and 4 °C. The chloroform fraction of lipophilic compounds was pipetted (about 0.5 mL) and evaporated in a vacuum concentrator (Eppendorf 5301, Hamburg, Germany) and dissolved again in 200 µL of methanol. A volume of 200 µL of water was added to obtain a 1/1 (v/v) of methanol/water and the sample was then filtered through 0.2 µm nylon membrane filters (Centrifugal Filters, WVR International, PA, USA) and placed into glass vials. The methanol/water fraction (fraction of polar compounds) was also filtered as described before and placed into glass vials.

Four carrots were selected for the optimisation procedure and the STDs were added to reach a final concentration of 0.02 µg mL^−1^ of phenolic acids, 0.1 µg mL^−1^ of vitamins and most of the flavonoids, and 0.2 µg mL^−1^ of (−)-epigallocatechin. Equal amount of carrots were used as control samples (without the addition of STDs) for the detection of the endogenous level of the substances used as STDs.

Repeatability of the extraction was calculated as the % relative standard deviation (RSD) of the peak intensity of the spiked STDs from six independent extractions. The analytical repeatability of the LC–MS was also measured as the % RSD of the measurements from one extract injected six times.

### 2.4 Metabolomics analysis

A total of 140 carrot samples (ten biological replicates from each field) were extracted as described above and analysed by UHPLC-MS on an UltiMate® 3000 Rapid Separation LC system (Dionex Corporation, California, USA) coupled to a Bruker micrOTOF II mass spectrometer (Bruker Daltonics, Germany) in a randomized way. Prior to analysis the extracts were conditioned at room temperature (20 °C) and separated using an Acquity UPLC BEH C_18_ column (100 mm × 2.1 mm, 1.7 µm particle size) coupled to an Acquity UPLC BEH C18 VanGuard, 5 mm × 2.1 mm, 1.7 µm particle size pre-column (Waters, Elstree, UK). The LC conditions were: injection volume 20 µL; autosampler temperature 10 °C; column temperature 25 °C; solvent flow 0.270 mL min^−1^.

Each fraction (methanol/water and chloroform) was run under three conditions: positive and negative electrospray ionization modes (ESI+ and ESI− respectively) with acidic mobile phases and in ESI− mode with a non-buffered neutral mobile phase.

Solvent A was 5% methanol, 0.2% formic acid in water and solvent B was 0.2% formic acid in methanol for ESI+ and ESI−. In addition, in ESI− mode, a non-buffered neutral mobile phase was also used as it proved to be necessary to detect or to improve the signal of some flavonoids and phenolic acid compounds. In both, ESI+ and ESI− modes, a linear solvent gradient was applied over 15 min from 100% solvent A to 100% solvent B, which was maintained for 15 min before re-equilibration with 100% A for 5 min. Every sample was injected once. The LC system was controlled by HyStar chromatography software (version 3.2) (Bruker Daltonic GmbH).

The MS conditions were as follows: acquisition mode ESI+ and ESI−, full scan 50 to 1000 *m*/*z*; capillary voltage +4.5 kV for ESI+ and −4.5 kV for ESI−; drying gas flow 10 L min^−1^; drying gas temperature 210 °C; nebulizing gas pressure (nitrogen) 1.7 bars. The mass accuracy was <1 ppm and the mass resolution >20,000 according to the tuning that was performed before the experiments. The spectral acquisition rate was 5 Hz and the vacuum pressure was approximately 4.2E−07 mbar. To ensure accurate mass measurements, 10 mM sodium formate solution in isopropanol/water (1:1) was injected at the beginning of each chromatographic run and used as internal calibrant.

For quality control (QC), mixtures of standards and mixtures of all carrot samples collected (two hundred and ten carrot samples or fifteen biological replicates from each field) were included in the injection sequence (every 10 samples). This injection was used for monitoring performance of the LC–MS system but not for correction purposes.

### 2.5 Data processing

After data acquisition, raw data was pre-processed using a bucketing approach in ProfileAnalysis V. 2.0. (Bruker Daltonics GmbH, Bremen, Germany). The peak detection algorithm 'find molecular features' was applied to the data under these parameters: S/N threshold 2; correlation coefficient threshold 0.7; minimum compound length 10 spectra; smoothing width 2. Advanced bucketing was performed in a time range from 0.5 to 20 min and in a mass range from 60.5 *m*/*z* to 850.5 *m*/*z* and using Δ RT = 0.3 min and Δ *m*/*z* = 5 mDa. The data was recalibrated automatically using automated post-run calibration by injection of sodium formate solution. Each dataset was normalized to the sum of bucket values in analysis. This means that bucket intensities in each analysis are normalized to the total intensity in the respective analysis. This normalization technique has the advantage that it can compensate for inaccuracies caused by injection (Wei et al., 2015).

### 2.6 Statistical analysis

Datasets were exported to SIMCA-P v12.0 software (Umetrics AB, Malmö, Sweden) for multivariate data analysis. All variables (molecular features) were Pareto scaled. Principal Component Analysis (PCA) was performed initially for unsupervised multivariate analysis. For predictive analysis, orthogonal projections to latent structures-discriminant analysis (OPLS-DA) models were built. The performance of all the models was evaluated by their explained variation (*R^2^X* for PCA and *R^2^Y* for OPLS-DA) and predictive ability (*Q^2^*). An internal sevenfold cross-validation was used to determine the significant components of the models and thus minimize overfitting. All OPLS-DA models are presented with the number of components based on the predictive performance from the internal cross-validation as suggested by the Simca-P+ software.

Initial OPLS-DA models were built on training sets containing two-thirds of the samples (randomly selected) and with molecular features selected based on a Variable Importance in the Projection (VIP) score >1. VIP scores summarize the importance of the x-variables for the model as a whole and molecular features with VIP values larger than 1 point to variables with large importance for that part of the model.

In addition to the internal cross-validation, they were also validated using the datasets (test sets or external validation sets) from the remaining one-third of the samples.

Further models were built removing the variation due the specific harvest year. These models were developed using a training set of randomly selected samples (two-thirds of the entire datasets) or with the deliberate removal of data from a given harvest year. Test sets with the remaining samples were then used for external validation of the resulting OPLS-DA models.

Predictions using the validation test sets allowed the calculation of sensitivity and specificity of the models. Additional models were built without using the cultivar Namur and then using those data for model validation. Unrelated samples from a similar environment or close distance (field codes 1a, 1b, 3a and 3b from Table S1 in Supplementary material) were also used for validation purposes.

Receiver Operating Characteristic (ROC) curves were computed to display the trade-off between sensitivity and specificity (Alonso et al., 2015, Eng, 2014). Sensitivity is defined as the percentage of objects belonging to a given class, which are correctly identified by the model (true positives), while specificity is the percentage of objects foreign to the modelled class (belonging to the other category) that are classified as foreign (true negatives). The Area Under the ROC curve (AUC) defines the overall ability of the test. An ideal model would have an AUC of 1.00 (i.e. when there is complete separation of the two agricultural production systems).

Potential candidates for discriminant markers were selected using the *S*-plot, which is a scatter plot of the covariance and correlation values of the loading profiles generated by the OPLS-DA models (Wiklund et al., 2008). The two tails of the *S*-plots contain the variables most contributing to differentiate the two groups; therefore those were selected to extract the potential discriminant markers. Candidates for discriminant markers were also selected by VIP values of the OPLS-DA models where a value higher than 1 was considered as the threshold. Twenty-four variables responsible for the discrimination of the two groups were subjected to Student's *t* tests. To do that, SYSTAT 13 software package (Systat Software GmbH, Erkrath, Germany) was used. Bonferroni-corrected *p*-values were also applied to avoid excessive false discovery rate due to multiple hypothesis testing (Broadhurst & Kell, 2006).

### 2.7 Metabolite identification

Metabolites were identified based on a comparison of retention time and mass spectrum to an authentic standard analysed under identical conditions following guidelines of the Metabolomics Standards Initiative (level 1) (Sumner et al., 2007).

Elemental compositions of putative structures were calculated using the SmartFormula tool from the DataAnalysis 4.0 software (Bruker Daltonics GmbH, Bremen, Germany), which provides a ranking according to the best fit of measured and theoretical isotopic pattern within a specific mass accuracy window. The quality of the isotopic fit was expressed by the mSigma-value. For level 2 identification (Sumner et al., 2007), a number of databases were used to search for the structural identity of the metabolites and these included; the human metabolome database (http://www.hmdb.ca/), the Kegg ligand database (http://www.genome.jp/kegg/ligand.html), the BiGG database (http://bigg.ucsd.edu/), the PubChem database (http://pubchem.ncbi.nlm.nih.gov/), the LIPID MAPS database (http://www.lipidmaps.org/), the MassBank database (http://www.massbank.jp) and the Metlin database (http://metlin.scripps.edu).

## 3 Results

### 3.1 Analytical performance

**Fig. 1:**
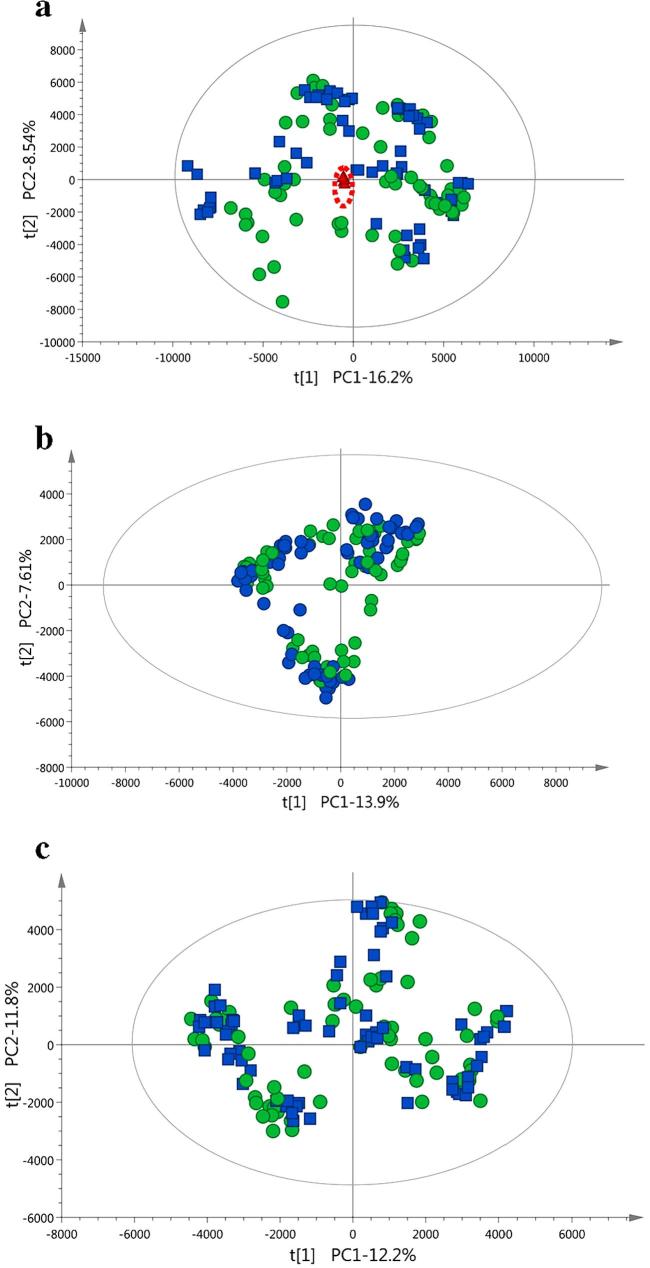
(a–c) PCA score plots of the first principal component (t1) versus the second principal component (t2) from the combined fraction’s (methanol/water and chloroform) mass spectra of the entire set of carrots under the (a) Electrospray ionization (ESI)+ mode. Quality control (QC) clusters (filled triangles) are highlighted within the ellipses. (b) ESI− mode and (c) ESI− with acid in the mobile phase. Organic samples (filled circles), conventional samples (filled squares). (For interpretation of the references to colour in this figure legend, the reader is referred to the web version of this article.)

The average repeatability standard deviation of the extraction method was 6%. PCA analysis was performed on the pooled QC samples to assess the stability of the UHPLC–MS system. The PCA analysis showed a tight clustering of QC samples, indicating stability and reproducibility during the sequence (Fig. 1a). Also, five representative peaks from the QC samples in each ionization mode covering a range of intensities and retention times were selected following the approach developed by Gika, Theodoridis, Earll, and Wilson (2012) as described in Table S3 (Supplementary material). The retention time shift ranged from 0.01 to 0.06 min, the mass error ranged from 0.10 to 4.49 mDa and the relative standard deviations of peak areas ranged from 1.2 to 12.7% verifying the stability of the whole analysis (Table S3 in Supplementary information).

### 3.2 Principal component analysis

**Fig. 2:**
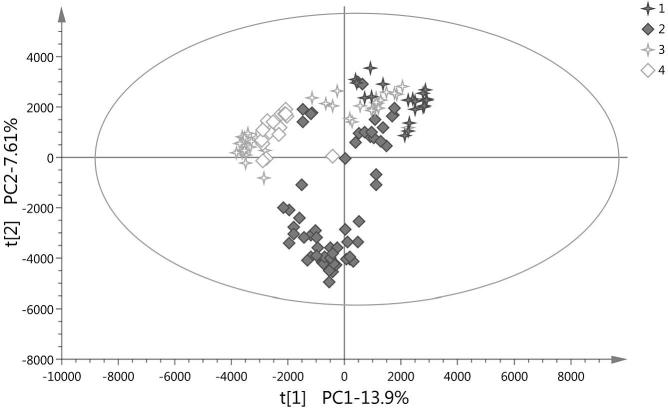
PCA score plots from the combined fraction's mass spectra using the Electrospray ionization (ESI)− mode (as Fig. 1b). The first and the second principal components (t1 and t2) are shown. Each year is represented with a different symbol. In picture legend 1: year 2005; 2: year 2006; 3: year 2007; 4: year 2008.

To investigate the main variance amongst the samples and to detect outliers, PCA was performed using each dataset separately: ESI− with a non-buffered mobile phase; ESI+ and ESI− with acid in the mobile phases. The PCA analysis highlighted the potential effect of the production year on the carrot metabolome (Fig. 2). The first two principal components explained 22% of data variation and showed clustering of the samples according to the year of harvest. The same pattern was observed in every dataset. The data was subjected to supervised multivariate data analysis to build models for classification of (future) samples.

### 3.3 Predictive modelling

Initially, one-third of the samples were randomly selected and set aside as a validation test set. The remaining samples (training set) were used to build the prediction models and to determine the differences in the metabolic fingerprint between conventional and organic samples. Of all the variables in the dataset, a large number are likely to be random variation and do not contribute to the distinction of the two agricultural systems. Scores of VIP > 1 generally represent those metabolites carrying the most relevant information for class discrimination (Umetrics, 2008). These variables were kept from the dataset and OPLS-DA models were built to distinguish samples from different production systems (Table 1, models 2–5). The resulting models were able to distinguish between organic and conventional carrots with high classification rates in the case of ESI+, ESI− and all the datasets combined (Table 1, models 2, 3 and 5). Supervised models carry a higher risk of overfitting and need to be validated. Classification rates were obtained from the internal cross-validation, but further external validations were performed using samples (validation test data sets) that were not used for the construction of the models. Correct classification rates of the non-modelled data (validation test data set) varied from 77.3% to 83.8%. The highest value was obtained when all the datasets from the different ionization modes were combined. If however, a data set representing the harvest from one year was left out of the training set and used as the validation test set, classification rates decreased to values as low as 48.3% (Table 1, models 6–9).

**Table 1:** Summary of OPLS-DA models for the distinction of carrot samples from organic and conventional agricultural systems and production year.

Model number	Classification based on	Ionisation Mode	Variables	Number of components (predictive component + orthogonal in X component + orthogonal in Y component)	R^2^Y	Q^2^ (cum)	Classification rate (Internal cross-validation)	Validation Samples (Test set)	Classification rate (external validation)
1	Year	All	11451	3 + 2 + 0	0.911	0.916	100%	–	–
*Refined datasets based on excluding variables with non-significant contribution (VIP < 1)*									
2	C vs O	ESI+	4083	1 + 5 + 0	0.972	0.561	100%	Random*	80%
3	C vs O	ESI−	1462	1 + 4 + 0	0.907	0.516	97.7%	Random*	77.3%
4	C vs O	ESI−a	1478	1 + 2 + 0	0.752	0.408	94.7%	Random*	83.7%
5	C vs O	All	20622	1 + 4 + 0	0.855	0.641	100%	Random*	83.8%
6	C vs O	All	20622	1 + 5 + 0	0.89	0.34	98.32%	2005	60%
7	C vs O	All	20622	1 + 6 + 0	0.911	0.725	100%	2006	48.3%
8	C vs O	All	20622	1 + 3 + 0	0.868	0.548	98.98%	2007	57.5%
9	C vs O	All	20622	1 + 3 + 0	0.841	0.618	98.31%	2008	55%

*Refined models based on excluding variables that contribute to the production year*									
10	C vs O	All	13250	1 + 4 + 0	0.95	0.655	100%	Random*	100%
11	C vs O	All	13250	1 + 3 + 0	0.961	0.754	100%	2005	88.2%
12	C vs O	All	13250	1 + 4 + 0	0.939	0.733	100%	2006	81.7%
13	C vs O	All	13250	1 + 4 + 0	0.982	0.781	100%	2007	75.7%
14	C vs O	All	13250	1 + 3 + 0	0.957	0.761	100%	2008	100%
15	C vs O	All	13250	1 + 4 + 0	0.964	0.733	100%	Cultivar Namur	90%
16	C vs O	All	13250	1 + 4 + 0	0.966	0.756	100%	Close Environment	88.6%

* Validation samples randomly selected (one third of the entire dataset).

a Acid in the mobile phase; C: conventional samples; O: organic samples; R^2^Y: explained variation; Q^2^(cum): predictive ability; VIP: Variable Importance in the Projection; ESI: electrospray ionization mode.

**Fig. 3:**
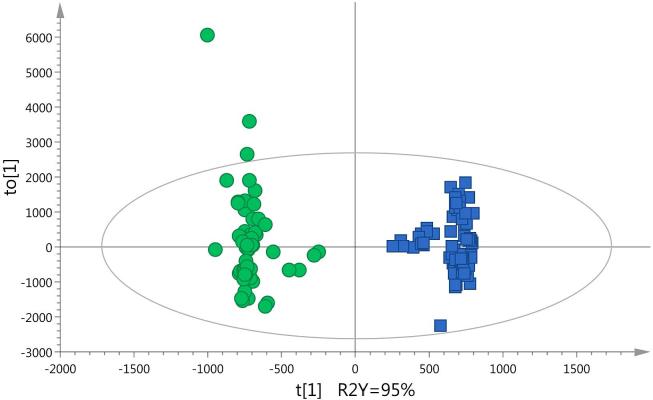
Score plot of OPLS-DA of model 10 (Table 1). The first predictive component (t_1_) and the first orthogonal component (t_o1_) are shown. R^2^Y: explained variation. Ellipse Hotelling’s T2 (95%). Organic samples (filled circles), conventional samples (filled squares). n = 130 (included validation samples/test set). (For interpretation of the references to colour in this figure legend, the reader is referred to the web version of this article.)

Fig. 2 shows clustering of the samples according to production year. This is visible between years 2005 and 2008 along the first principal component (horizontal axis). By using a supervised technique, OPLS-DA, and the combined datasets (different fractions and ionization modes), the production year of the samples was successfully distinguished with a high explained variation (*R^2^Y*) and predictive ability (*Q^2^_cum_*) (close to 1) (Table 1, model 1), confirming the influence of the production year on the carrot metabolome. Since discrimination of individual harvest years was not the objective of the study, variables with a VIP > 1 in the OPLS-DA model, and therefore, responsible for the classification of samples according to their production year, were subsequently excluded from the dataset. After removal of these variables, a model with a sensitivity (percentage of true positives or conventional samples correctly classified as conventional) and specificity (percentage of true negatives or organic samples correctly classified as such) of 100% was obtained. This model was built from a training set of randomly selected samples across the years (Table 1, model 10 and Fig. 3).

**Fig. 4:**
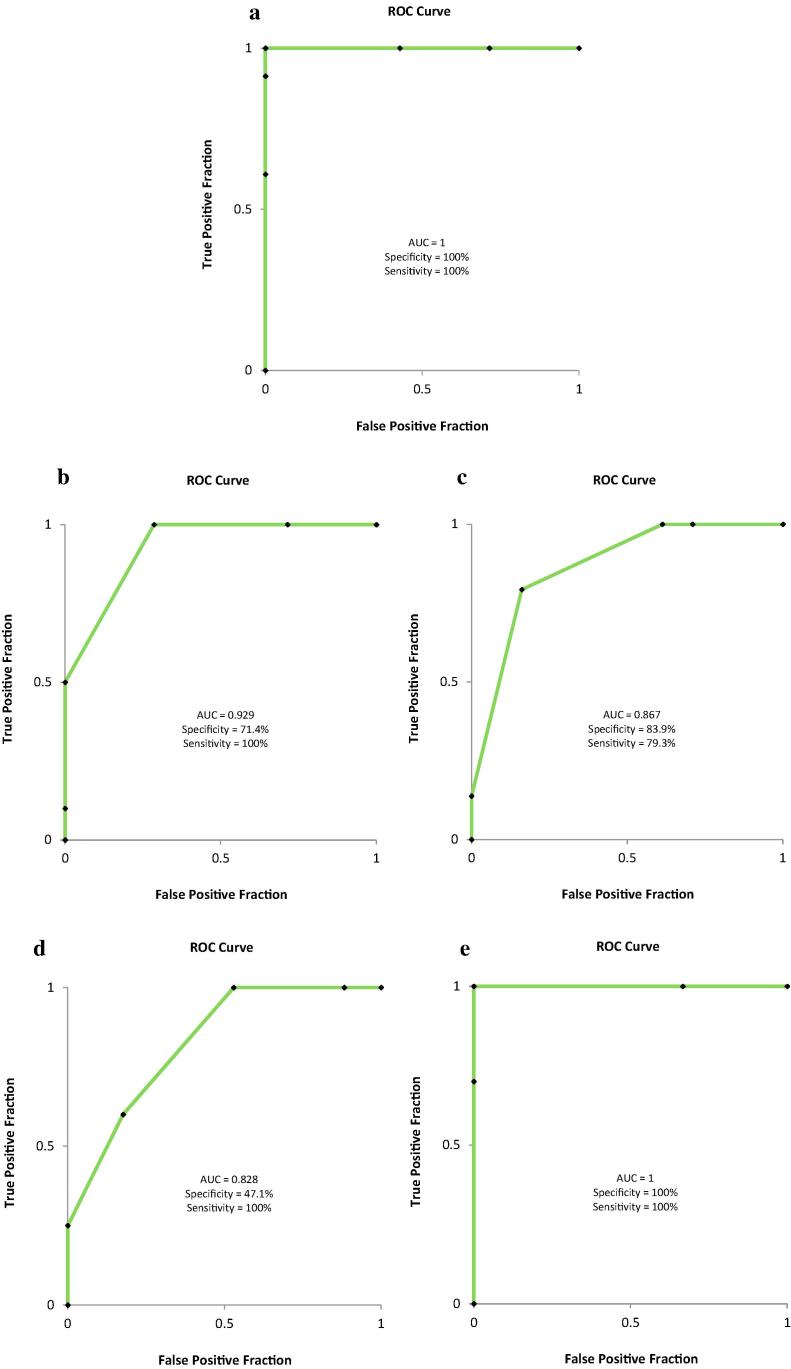
ROC curves of external validation of models 10–14 from Table 1. a: random samples used as validation set (Model 10); b: samples from 2005 as external validation test set (Model 11); c: samples from 2006 as external validation test set (Model 12), d: samples from 2007 as external validation test set (Model 13); e: samples from 2008 as external validation test set (Model 14). ROC = Receiver Operating Characteristic; AUC = Area Under the ROC Curve.

To test the robustness of this approach, external validation test sets consisting of samples from one harvest year were used to predict their agricultural origin using a model built with samples from the remaining harvest years. The four models obtained showed high correct classification rates (>75%) (Table 1, models 11–14) and appropriate sensitivity and specificity expressed as AUC (Fig. 4), except for the harvest of 2007, where specificity (or true negative rate) was 47.1% (Fig. 4d). Moreover, cultivar ‘Namur’ was also used as validation test set on models built from cultivar ‘Nerac’ to predict class membership of a different cultivar (‘Namur’). The classification rate obtained was 90% (Table 1, model 15). Additionally to the 'Namur' carrots, samples from 2005 (Nerac), coming from a close-distance environment were also removed from the training sets and used as validation test sets to check the effect of the environment on the robustness of the model. The resultant model and prediction parameters were also high (Table 1, model 16).

The results obtained indicate that metabolomics using a multivariate analysis approach is a promising tool to discriminate between agricultural systems considering the AUC values were higher than 0.7. The OPLS models developed take into account a wide range of other factors such as different years, varieties and geographical locations as well as different fertilization and phytochemical treatments (Table S1 and Fig. S1 in Supplementary material).

### 3.4 Metabolites of organic and conventional samples

OPLS-DA models showed a clear separation between conventional and organic samples (Fig. 3). The S-plot showing the contribution of the variables, together with the VIP scores, was used to lead the identification of potential markers of the agricultural system used. The 24 metabolites that contributed most to the metabolite signature were selected to be further studied from which a total of 15 could be identified (levels 1–3; Sumner et al., 2007) (Table 2). They included several carbohydrates, l-arginine, chlorogenic acid, and citric acid. Compounds that were putatively identified included a carbohydrate, fatty acid esters, a jasmonate derivative, and glycosylated terpenes.

**Table 2:** Markers of agricultural system discrimination in carrots. *P* values (uncorrected) are calculated from univariate statistics (unpaired Student’s *t* test).

RT	Polarity Molecular Ion	Fraction	*m*/*z*	Observed Empirical formula	Δppm	Fragment ions				Metabolite	Ratio organic/conventional^a^	*P* value (uncorrected)
						*m*/*z*	Formula	Δppm				
*Identified compounds (level 1)*												
1.3	+	MW/C	233.0632	C_7_H_14_O_7_Na (M+Na)	0.4	193.0707 157.0495	C_7_H_13_O_6_ C_7_H_9_O_4_		0 0.6	Sedoheptulose	1.7	1.17E−04
1.3	+	MW	365.1054	C_12_H_22_O_11_Na (M+Na)	1.3	325.1124 163.0601	C_12_H_21_O_10_ C_6_H_11_O_5_		0.8 1.4	Dissaccharide	1.3	1.02E−04
1.5	+	MW	175.1190	C_6_H_15_N_4_O_2_ (M+H)	1.1	116.0706	C_5_H_10_NO_2_		1.1	l-Arginine	2.0	2.51E−04
1.5	+	MW/C	432.1712	C_15_H_30_NO_13_ (M+NH_4_)	0.3	127.0390	C_6_H_7_O_3_		0	Arabinofuranosyl-[a-l-arabinofuranosyl)]-l-arabinose (Trisaccharide)	1.5	4.86E−04
1.9	+	MW	215.0162	C_6_H_8_O_7_Na (M+Na)	2.3	175.0237 147.0288	C_6_H_7_O_7_ C_5_H_7_O_5_		2.7 3.0	Citric acid	1.5	1.93E−06*
7.0	+	MW/C	355.1024	C_16_H_19_O_9_ (M+H)	0.3	163.0390	C_9_H_7_O_3_		0.1	Chlorogenic acid	1.5	8.96E−03
1.2	–	MW/C	179.0561	C_6_H_11_O_6_ (M−H)	1.4	119.0350 89.0544	C_4_H_7_O_4_ C_3_H_5_O_3_		0.7 6.7	Monosaccharide	1.2	3.23E−03
1.3	–	MW/C	209.0660	C_7_H_13_O_7_ (M−H)	3.4	119.0350	C_4_H_7_O_4_		2.7	Sedoheptulose	1.7	3.17E−03
1.3	–	MW/C	245.0434	C_7_H_14_O_7_Cl (M+Cl)	1.6	209.0660 119.0350	C_7_H_13_O_7_ C_4_H_7_O_4_		1.2 2.1	Sedoheptulose	1.6	2.00E−03
7.0	–	MW	353.0878	C_16_H_17_O_9_ (M−H)	0	191.0561 179.0350 161.0244 135.0452	C_7_H_11_O_6_ C_9_H_7_O_4_ C_9_H5O_3_ C_8_H7O_2_		0.0 7.3 4.8 2.2	Chlorogenic acid	1.4	8.07E−03

*Putatively annotated compounds or compound class (level 2 and 3)*												
17.0	+	MW/C	518.3280	C_29_H_46_NO_4_Na_2_ (M+2Na−H)	1.7					Fatty acid ester	2.0	4.90E−04
6.8	–	MW/C	391.1610	C_17_H_27_O_10_ (M+FA−H)	2.4					Terpene glycoside	6.0	1.69E−09*
8.1	–	MW	373.1504	C_17_H_25_O_9_ (M+FA−H)	3.3					Terpene glycoside	2.4	5.03E−08*
8.9	–	MW	507.2083	C_22_H_35_O_13_ (M−H)	0.5					Oligosaccharide	5.5	1.11E−07*
9.1	–	MW	369.1555	C_18_H_25_O_8_ (M−H−H_2_O)	0.0	247.1187 141.0557	C_11_H_19_O_6_ C_7_H_9_O_3_	1.9 1.7		12-Hydroxyjasmonic acid glucoside	3.9	1.46E−08*

*Unknown compounds (level 4)*												
1.3	+	MW/C	413.1261							Unknown	2.8	8.49E−06
1.4	+	MW/C	397.1310							Unknown	1.9	9.67E−04
15.7	+	C	406.0746							Unknown	0.5	6.37E−06
1.2	–	MW/C	119.0374							Unknown	1.2	3.17E−03
1.7	–	MW	719.2690							Unknown	0.8	8.55E−03
6.6	–	MW	315.0723							Unknown	2.0	5.90E−04
9.5	–	C	325.1078							Unknown	0.6	4.38E−03
11.4	–	MW	555.1140							Unknown	0.7	3.65E−02
17.9	–	C	452.2785							Unknown	1.3	1.85E−02

Δppm: is the difference in exact mass between the measured value and the theoretical value. RT: retention time; MW: methanol/water fraction; C: chloroform fraction.

a Ratio was calculated as peak area ratio from the arithmetic mean values of each group.

* Significant *p*-values after Bonferroni corrections (*p* < 3.77E−06).

Five of the 24 metabolites showed significant differences between systems using a univariate approach after Bonferroni corrections (*p* value < 3.77E−06) considering the total number of variables as the number of independent tests.

## 4 Discussion

The influence of organic and conventional agricultural practices on the endogenous chemical composition of crops is disputed in the literature. The purpose of this study was to assess if there was an effect of the agricultural practice on the crop's metabolome and to assess the potential of metabolomics approaches in organic food authentication.

The aim of any metabolomic study is to screen and monitor many molecules over a diverse chemical spectrum and concentration range. However, different ionization and extraction requirements of different metabolites limit the analytical techniques used in metabolomics and make the detection of all metabolites present in a biological organism using a single methodology almost impossible. Since the aim is to extract and detect a large number of molecules, both ESI− and ESI+ were used to profile the metabolites of carrots. The results indicated that the two modes were complementary to each other. Under these conditions all the standards used for method optimisation could be detected in ESI- and/or in ESI+ forming [M−H]^−^, [M+CH_2_O_2_]^−^, [2M−H]^−^, [2M−H+Na]^−^, [M+H]^+^, [M+Na]^+^ [2M+H]^+^ and/or [2M+Na]^+^ species. The extraction method used in this study proved to be highly repeatable and had a high metabolic yield.

In metabolic fingerprinting, large amounts of data (high number of variables) are produced. For discriminative and prediction metabolomics, models that associate metabolite data with membership of sample classes are built (supervised methods). OPLS-DA methods can be applied to visualize variations between sample groups and to define the discriminating performance of the variables. PCA analysis failed to separate samples based on the production system (Fig. 1). This is likely due to the fact that PCA is an unsupervised technique (separates the samples without class information), and the differences in metabolites between organic and conventional samples may be lower than those due to other factors such as year, geographical location or varieties studied. The two first principal components of the PCA applied on all the carrot samples explained 22% of the overall existing variability and the samples clustered partially by production year (Fig. 2). Recent metabolomics studies of organic and conventional cabbages, potato, wheat, tomatoes and peppers also reported that the year of production had stronger influence on the metabolomic fingerprint compared to the type of production system (Kessler et al., 2015, Mie et al., 2014, Novotná et al., 2012, Shepherd et al., 2014). The data was subjected to supervised multivariate data analysis to remove the large inter-year variation. Large numbers of variables in the dataset are likely to be random variation, which can reduce the performance parameters of the model. To reduce the random variation and isolate the information important for the classification of production systems two approaches were taken. First, variables that had a non-significant contribution to the model were removed from the datasets using as criteria VIP < 1. This approach is thought to increase the overfitting risk of the models since false-positives variables are enriched in the new datasets created (Mie et al., 2014). This is confirmed by the lower predictive ability (*Q^2^*) of the models (Table 1 models 6–9, and Fig. S2 in Supplementary material). If instead, variables that contributed to the classification of samples based on production year were removed from the datasets, predictive ability, specificity and sensitivity values of the models generally improved (Table 1, models 10–14 and Fig. 4). This confirms that in our study, the production year is the most important source of variation in the crop's metabolome. It also highlights the importance of refining the models to select the variables responsible for the classification of samples into production systems. In our case, a large set of samples from different years, varieties, and locations ensured that the models built kept the imprint left in the metabolome by the production system.

ROC curves are a graphical summary of the performance of a binary classifier and can be used to visualize the tradeoff between sensitivity and specificity in the classification (Fig. 4). In food authentication settings both sensitivity and specificity can be important. In the organic authentication context, it is very important that samples labeled as organic are classified as organic (true negative or specificity) since most of the fraud cases may be caused by the wrong labelling of conventional products as being organic. One could, therefore, choose models characterized by elevated specificity to address this problem (Mie et al., 2014, Oliveri et al., 2011). The classification rates obtained in this study, which were externally validated by using datasets representing whole harvest years, are comparable to those reported by Mie et al. (2014), who discriminated organic and conventional cabbages. However, their dataset was limited to samples taken from a controlled trial conducted over two years; the model based on the 2008 harvest had indeed a high prediction ability of samples taken in 2007 but not *vice versa*. Novotná et al. (2012) built models for discriminating organic and conventional tomatoes and peppers with prediction abilities higher than 80%. Nevertheless, they were calculated using a leave-one-out approach; no external data sets were used for validating the prediction models. In maize, a three-year study found that genotype and environment were the major contributors of metabolite differences (Röhlig & Engel, 2010). Only three compounds were consistently different between organic and conventional crops over the years. In wheat, Zörb, Niehaus, Barsch, Betsche, and Langenkämper (2009) found no differences in matured grain between organic and conventional crops taken in one year, whereas Bonte et al. (2014) found differences between both production systems only when different cultivars were analysed separately. In this case, they concluded that the influence of cultivar was higher than that of the production system. Similarly, Kessler et al. (2015), using wheat grains grown over three years found only differences due to the production system as the crops were individually analysed each year because of the massive influence of the factor year on the metabolomic fingerprint. Chen and Harnly, 2010, Shepherd et al., 2014, and Vallverdú-Queralt et al. (2011) showed that the production system had an influence on the metabolite profiles of tomatoes, tomato-derived products, red grapefruit and potatoes but for the latter differences associated with the production systems were much lower and less consistent compared with the fertilizer effects found. The major strength of our study is that we found a systematic and consistent influence of the production system on the crop's metabolome in two varieties of carrots collected from farmers using different phytochemical and fertilization approaches and in different locations of Belgium over a period of four years. This highlights the potential of using metabolomics for authentication of agricultural origin of organic products. It also becomes evident that an extensive dataset, which includes different growing areas, plant varieties, fertilisation practices and production years to account for natural variation is necessary to develop statistical models with high rates of correct classification (Cubero-Leon et al., 2014).

Although the identification and quantification of the metabolites responsible for the classification were not the aim of this study, efforts were made to get deeper insights into the metabolic differences. Twenty-four variables with high VIP score values and high covariance and correlation values of the loading profiles were tentatively identified by isotope and fragmentation patterns and, when available, matching of the retention times, isotope and fragmentation patterns to standards. The differences in production system seen in the multivariate data analysis are reflected in the data from the univariate analysis (Student’s *t* test, Table 2). Bonferroni corrections were made to take account of multiple comparisons (Broadhurst & Kell, 2006). Following this approach the concentrations of five metabolites were significantly different (Table 2, compounds with p < 3.77E−06). Interestingly, markers related to carbohydrate metabolism and plant defence mechanisms were identified as being the metabolites mainly involved in the differences between both types of agricultural systems. Similar effects related to the glucose and organic acid metabolism are known from other studies. A metabolomics study on potatoes grown under conventional and organic practices also reported higher levels of certain sugars (Shepherd et al., 2014) in the organic crops. Mechanic removal of foliage in the organic potatoes may have been the cause of some differences in the carbohydrate transport to tubers prior to harvest. There are many reports that highlight the importance of high levels of sugars in plant resistance to diseases caused by fungal pathogens (Morkunas & Ratajczak, 2014). However, Maggio et al. (2008) found that the carbohydrate content, with the exception of fructose, was not affected by the cultivation regime. On the other hand, they found that over-fertilization significantly inhibited the biosynthesis of sucrose, fructose and glucose. In our study, sedoheptulose content seems to be higher in organic carrots than in conventional ones. The presence of sedoheptulose has been described before in carrots by Soria, Sanz, and Villamiel (2009).

Nitrogen in conventional fertilizers is readily available to plants whereas only a small percentage of N (10–15%) is directly available from organic manures (Shepherd et al., 2014). This high availability of N in conventional crops could also be the cause of high levels of amino acids reported in the literature (Capuano et al., 2013, Shepherd et al., 2014, Zörb et al., 2009). However, in this study, arginine showed a trend of upregulation in organic crops. As stated by Bradi, Zolla, Bakker, Manter, and Vivanco (2013), soil microbiomes can also impact the metabolome of plants. The use of pesticides is likely to alter the soil microbiome and therefore, could indirectly be another cause for the differences seen in the carrot's metabolome of organic and conventional crops (Mie et al., 2014, Ruzicka et al., 2012). Under conditions of limited plant nutrient availability, as is the case in organic crop husbandry, certain processes of the secondary plant metabolism, such as plant defence, stress tolerance and ripening are expected to be more pronounced (Capuano et al., 2013). Phenolic acids, such as chlorogenic acid is a typical secondary metabolite. Data reported in the literature for chlorogenic acid contents are controversial. Our results are in line with a previous study by Vallverdú-Queralt et al. (2011) who found significantly higher concentrations of chlorogenic acid in ketchup produced from organically grown tomatoes. By contrast, Caris-Veyrat et al. (2004) found that chlorogenic acid contents were lower in organically grown tomatoes. In peppers, Novotná et al. (2012) found phenolic acids and citric acid as discriminant markers of organic and conventional practices. In plants, a large fraction of terpenoids is present as non-volatile glycosides. Terpenoids are thought to protect plants that produce them by deterring herbivores. Attack from insects may stimulate the production of certain secondary compounds in carrots as a protection mechanism. Long term stress from insect attack by carrot psyllid (*Trioza apicalis*) as well as low nitrogen fertilisation levels had an enhancing effect on terpene accumulation (Seljåsen et al., 2013). Another secondary metabolite putatively identified in our study was a jasmonate derivative. Jasmonic acid and its derivatives play important roles in controlling growth, development and responses to environmental changes in plants (Nakamura et al., 2011). The jasmonic acid signalling pathway plays a central role in plant defence against necrotrophic pathogens in herbivorous insects that affect roots (Carvalhais et al., 2013, Shrivastava et al., 2010), which could hypothetically explain the elevated levels observed in our study.

## 5 Concluding remarks

This study adds to the small, but growing number of reports on the use of ‘omics’ for food authentication purposes and highlights the potential of metabolomics approaches to differentiate between organic and conventionally grown crops. We have included several influencing factors in our experimental design, such as different locations, varieties and production years.

The results obtained indicate that the production system has a systematic influence on the carrot’s metabolome. With the use of OPLS-DA it is possible to classify samples according to agricultural practices and predict the origin of unknown samples. Markers related to carbohydrate metabolism and plant defence mechanism were identified as responsible for the differences between both agricultural systems. The samples analysed here were collected directly from the fields in order to ensure their authenticity and not from commercial settings where other influencing factors such as storage time and conditions could also have an influence in the metabolome.

A pan-European experimental study embedding a greater variability such as geographical locations with different pedo-climatic conditions and more cultivars could allow for the development of robust classification models to be ready for use in food authentication control practices.

## Conflict of interest disclosure

This research did not receive any specific grant from funding agencies in the public, commercial, or not-for-profit-sectors. The authors declare no conflicts of interest.
