# Supplementary material for: Metabolomics for organic food authentication: Results from a long-term field study in carrots
Source: Food Chem. 2018 Jan 15;239:760–70. doi: 10.1016/j.foodchem.2017.06.161 (PMC5611763; doi:10.1016/j.foodchem.2017.06.161)
Supplement: Supplementary data [file mmc1.docx]

| Field code | Agricultural system | Site | Year | Cultivar |
| --- | --- | --- | --- | --- |
| 1a | O | Waremme | 2005 | Nerac |
| 1b | C | Waremme | 2005 | Nerac |
| 2a | O | Borlez | 2006 | Nerac |
| 2b | C | Borlez | 2006 | Nerac |
| 3a | O | Waremme | 2006 | Namur |
| 3b | C | Waremme | 2006 | Namur |
| 4a | O | Éghezée | 2006 | Nerac |
| 4b | C 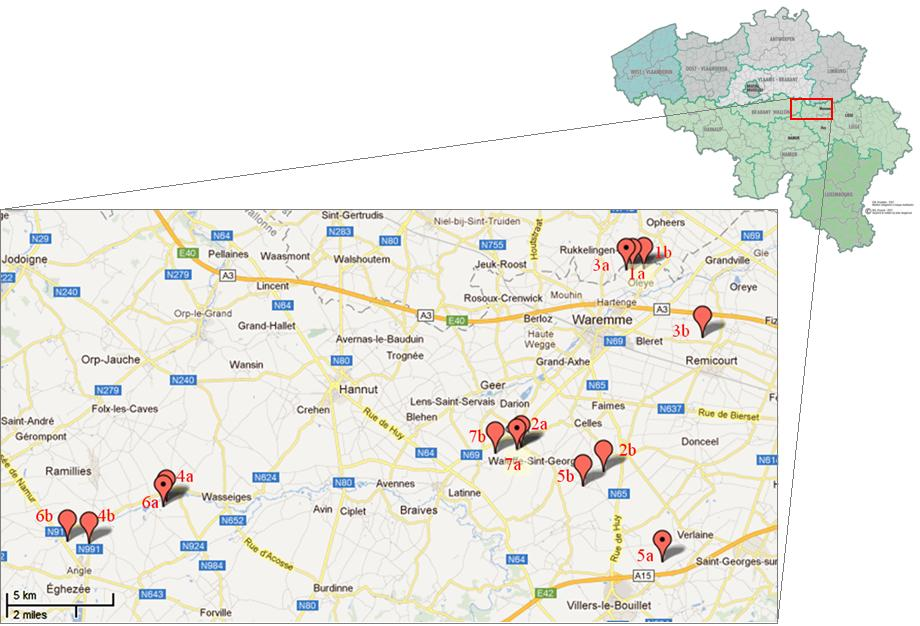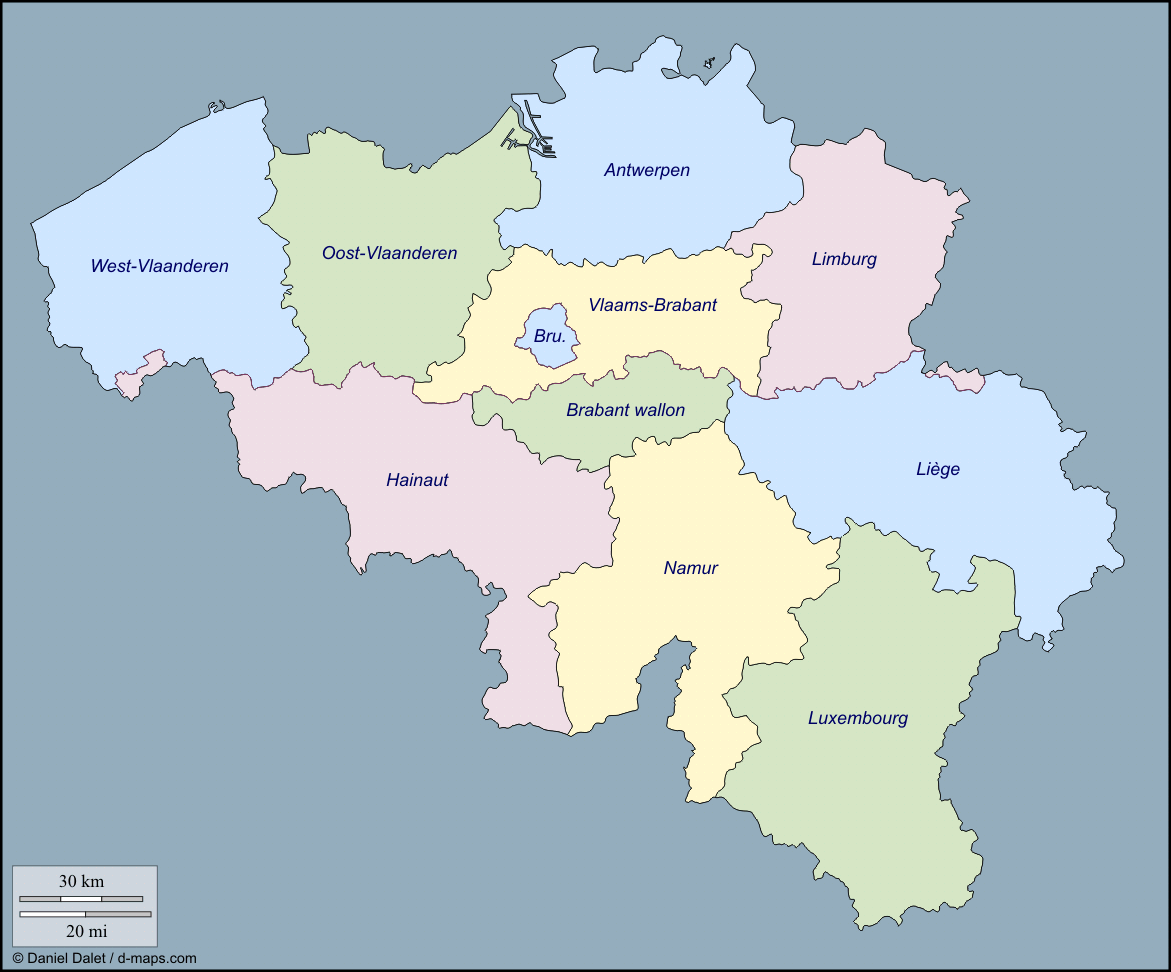 | Éghezée | 2006 | Nerac |
| 5a | O | Borlez | 2007 | Nerac |
| 5b | C | Borlez | 2007 | Nerac |
| 6a | O | Éghezée | 2007 | Nerac |
| 6b | C | Éghezée | 2007 | Nerac |
| 7a | O | Borlez | 2008 | Nerac |
| 7b | C | Borlez | 2008 | Nerac |

**Fig. S1** Geographical location of carrot field samples collected in 2005, 2006, 2007 and 2008. (Map data _ 2014 Google). O: organic farming,; C: conventional farming

**b**

**a**


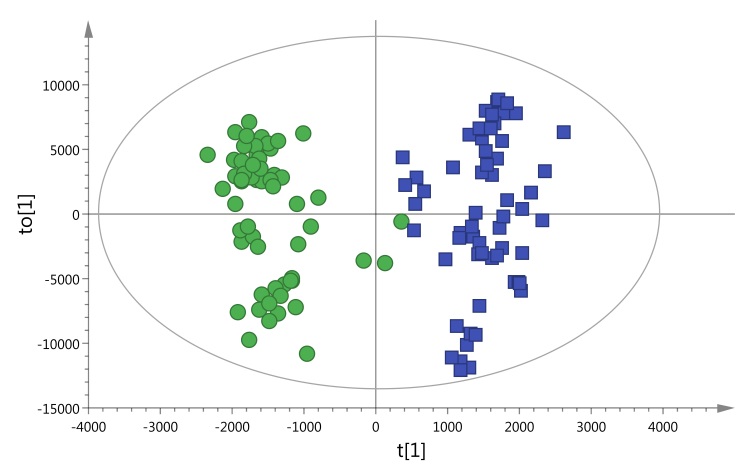
**
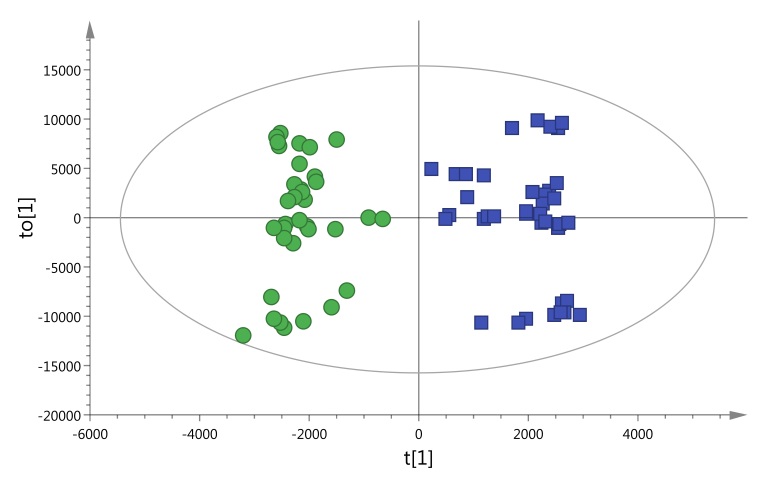
**

**d**

**c**


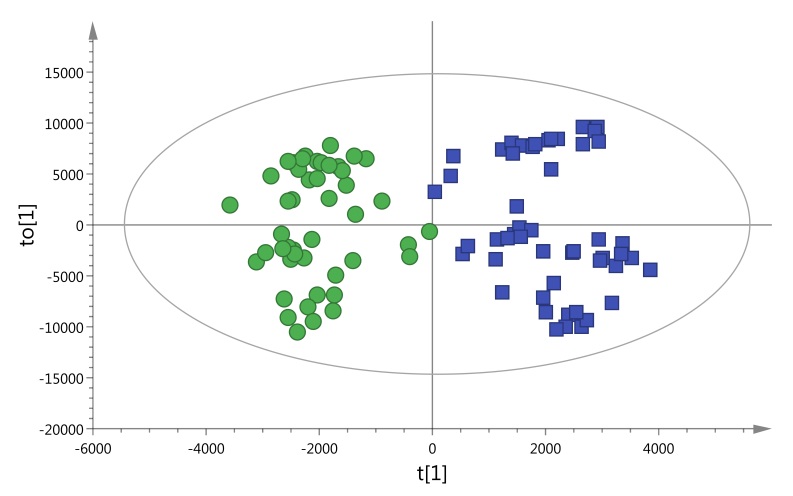

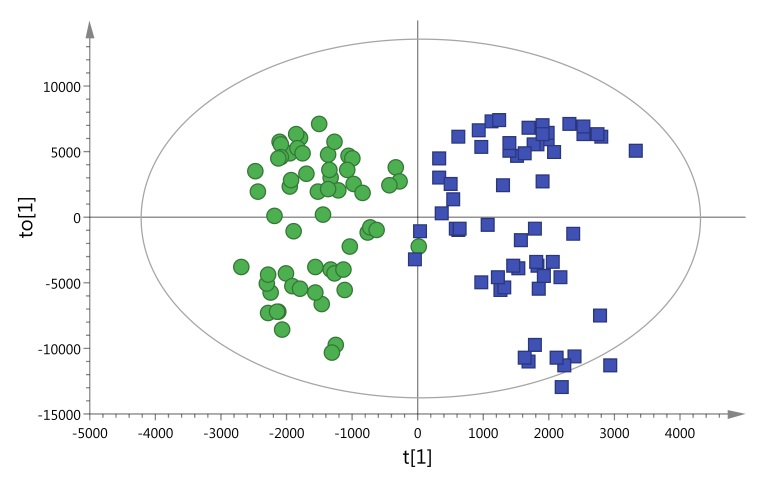


**Fig. S2** Scores plot of OPLS-DA models 6 (a), 7 (b), 8 (c) and 9 (d) from Table 1. The first predictive component (t_1_) and the first orthogonal component (t_o1_) are shown. Ellipse Hotelling's T2 (95%). Organic samples (filled circles), conventional samples (filled squares) are also coloured (Colour figure online). Model 6 had a specificity of 75% and a sensitivity of 44.4%. Model 7 had a specificity of 22.6% and a sensitivity of 74.2%. Model 8 had a specificity of 40.9% and a sensitivity of 76.5%. Model 9 had a specificity of 100% and a sensitivity of 10%.

**Table S1** Dates of sowing and harvest, fertilization practices and yields from the fields selected for this study.

| **Year** | **Field**  **code** | **Agricultural system** | **Sowing date** | **Harvest date** | **Yield**  (T ha^-1^) | **Fertilisation**  Commercial name and amount | **Phytochemicals**  Commercial name and amount | **Crop rotation** |
| --- | --- | --- | --- | --- | --- | --- | --- | --- |
| 2005 | 1a | O | 21/04/2005 | 21/10/2005 | 32 | Horpi/Syst (1350 Kg ha^-1^) | None | 2004: winter wheat; 2003: maize; 2002: spelt |
|  | 1b | C | 18/05/2005 | 28/09/2005 | 45 | 0/5/14 + 3 MgO (1500 Kg ha^-1^); Kiserite (200 Kg ha^-1^); N solution 27/0/0  (170 Kg ha^-1^) | Hurican (6.2 L ha^-1^); Afalon (0.45 L ha^-1^); Centium (0.2 L ha^-1^); Afalon (0.15 L ha^-1^); Dosanex (1 Kg ha^-1^); Top oil (0.5 L ha^-1^); Dosanex (0.5 Kg ha^-1^); Dosanex (1.5 Kg ha^-1^); Top oil (0.5 L ha^-1^); Horizon (1 L ha^-1^) | 2004: pea; 2003: sugar beet; 2002: winter wheat |
| 2006 | 2a | O | 01/05/2006 | 17/10/2006 | 48 | None | None | 2005: carrot; 2004: bean; 2003: winter wheat |
|  | 2b | C | 10/05/2006 | 09/10/2006 | 65 | N solution (37%); N.P.K. 0/8/18 (1000 Kg ha^-1^) | Linagan (0.4 L ha^-1^); Centium (0.180 L ha^-1^); Priglone (2 L ha^-1^); Dosanex (1.2 Kg ha^-1^); Dosanex (1.5 Kg ha^-1^); Dosanex (1.22 Kg ha^-1^); Afalon (0.1 L ha^-1^); Ortiva (1 L ha^-1^); Karate (0.1 L ha^-1^); Soufre (2.5 Kg ha^-1^); Magnesie (6.0 Kg ha^-1^); Geyser (0.5 L ha^-1^); Ortiva (1.0 L ha^-1^); Magnesie (6 Kg ha^-1^) | 2005: winter wheat; 2004: sugar beet; 2003: winter wheat and phacelie |
| 2006 | 3a | O | 11/04/2006 | 06/10/2006 | 45 | Chicken manure (5 T ha^-1^) | Not recorded | 2005:Winter wheat and phacelie |
|  | 3b | C | 15/04/2006 | 06/10/2006 | 68 | 18 N (400 kg ha^-1^); 46 P (400 kg ha^-1^); 30 K (600 kg ha^-1^); 25 MgO (100 kg ha^-1^) | Centium (0.19 L ha^-1^); Linuron (0.45 L ha^-1^); Linaron (0.2 L ha^-1^); Dosanex (1.2 Kg ha^-1^); Dosanex (2 Kg ha^-1^); Ortiva (1 L ha^-1^); Soufre (3 Kg ha^-1^); Karate Zeon (0.1 L ha^-1^); Magnesie (3 Kg ha^-1^) | 2005: winter wheat; 2004: sugar beet; 2003: flax and mustard |
|  | 4a | O | 17/05/2006 | 09/10/2006 | 55 | Fertifior (14/4/6) (3 T ha^-1^) | None | 2005: spinach; 2004: bean; 2003: winter wheat |
|  | 4b | C | 08/05/2006 | 24/10/2006 | 68 | N solution (39%) (39/0/0) (200 L ha^-1^); (0/11/16.5) (1 T ha^-1^) | Afalon (0.45 L ha^-1^); Centium (0.18 L ha^-1^); Dosanex (1.5 Kg ha^-1^); Dosanex (1.4 Kg ha^-1^); Dosanex (1.22 Kg ha^-1^); Ortiva (1.0 L ha^-1^); Karate (0.1 L ha^-1^); Soufre (2.5 Kg ha^-1^); Geyser (0.5 L ha^-1^); Ortiva (1.0 L ha^-1^); Soufre (2.5 Kg ha^-1^) | 2005: winter wheat; 2004: winter wheat; 2003: sugar beet |
| 2007 | 5a | O | 31/05/2007 | 01/10/2007 | Not recorded | Chicken manure (7 T ha^-1^) | Cuivre (3 kg ha^-1^); Soufre (4 Kg ha^-1^); Cuivre (3 Kg ha^-1^); Soufre (4 Kg ha^-1^); LithalgueH 400 (50 Kg ha^-1^) | 2006: barley; 2005: winter wheat; 2004: flax |
|  | 5b | C | 30/05/2007 | 01/10/2007 | Not recorded | Bulk (3/5/10 + 6 + 8) (2.3 T ha^-1^) | Linuron (0.5 L ha^-1^); Centium (0.15 L ha^-1^); Disonal (2 L ha^-1^); Dosanex (1 Kg ha^-1^); Dosanex (1.5 Kg ha^-1^); Ortiva (0.8 L ha^-1^); Geyser (0.5 L ha^-1^); Ortiva (1 L ha^-1^) | 2006: winter wheat; |
|  | 6a | O | 05/06/2007 | 03/10/2007 | Not recorded | Fertifior (14/4/6) (7 T ha^-1^) | None | 2006: bean; 2005: not recorded; 2004: sugar beet |
|  | 6b | C | 05/06/2007 | 03/10/2007 | Not recorded | None | Centium (0.15 L ha^-1^); Afalon (0.45 L ha^-1^); Disonal (2 L ha^-1^); Dosanex (1.5 Kg ha^-1^); Dosanex (2 Kg ha^-1^); Ortiva (1 L ha^-1^); Geyser (0.5 L ha^-1^) | 2006: winter wheat; 2005: winter wheat; 2004: sugar beet |
| 2008 | 7a | O | Not recorded | Not recorded | Not recorded | Not recorded | Not recorded | Not recorded |
|  | 7b | C | 09/05/2008 |  | 120 | i) 0/6/18 + 3 + 2 (1580 Kg ha^-1^); ii) N solution (39/0/0) (100 L ha^-1^); iii) Ammonium nitrate (27/0/0) (100 Kg ha^-1^) | i) Ouragan (4 L ha^-1^); ii) Centium (0.15 L ha^-1^); iii) Afalon (0.4 L ha^-1^); Sencor (0.093 L ha^-1^); Sencor (0.093 L ha^-1^); Afalon (0.2 L ha^-1^); Nativo (0.3 Kg ha^-1^); Wett 90 (0.25 L ha^-1^); Signum (0.750 Kg ha^-1^); Signum (0.750 Kg ha^-1^); Nativo (0.3 Kg ha^-1^); Difcor (0.5 L ha^-1^); Difcor (0.37 L ha^-1^) | Not recorded |

O: organic farming, C: conventional farming

**Table S2** Soil properties

| Year | Field Code | Particle size (%) | | | | | |  | | | | mg/100 g | | |  | cmol/kg | | | | | | | | | |
| --- | --- | --- | --- | --- | --- | --- | --- | --- | --- | --- | --- | --- | --- | --- | --- | --- | --- | --- | --- | --- | --- | --- | --- | --- | --- |
|  |  | Sand  >50 µm | Loam  2-50  µm | Loam  20-50  µm | Loam  10-20 µm | Loam  2-10  µm | Clay  <2 µm | pH  (H_2_O) | pH (KCl) | Org. Mat. % | C  % | Total N | Ammonium-N | Nitrate-N | C/N | K | Na | Cd | Cu | Fe | Hg | Pb | Se | Zn | Mn |
| 2005 | 1a | 10.3 | 76.9 | 52.5 | 17.3 | 7.1 | 12.8 | 7.4 | 7.3 | 2.1 | 1.2 | 124 | 1.78 | 31.90 | 21.15 |  |  | <0.50 | 14.9 | 20940 | <0.10 | 13.4 | <0.20 | 67.1 | 228 |
| 2005 | 1b | 12.6 | 74.9 | 48.8 | 17.6 | 8.5 | 12.5 | 7.6 | 7.8 | 2.4 | 1.4 | 144 | 2.04 | 46.02 | 19.44 |  |  | <0.50 | 13.1 | 17908 | 0.12 | 16.3 | <0.20 | 75.9 | 213 |
| 2006 | 2a | 14.7 | 68.5 | 53.4 | 13.3 | 1.8 | 16.8 | 7.3 | 7.0 | 2.1 | 1.2 | 135 | 0.50 | 2.14 | 8.89 | 0.99 | 0.30 | 0.18 | 15.9 | 20400 | 0.067 | 24.8 | 0.16 | 80.6 | 215 |
| 2006 | 2b | 11.7 | 70.9 | 52.6 | 13.0 | 5.3 | 17.4 | 7.4 | 7.2 | 1.7 | 1.0 | 115 | 0.52 | 2.37 | 8.70 | 1.01 | 2.48 | 0.35 | 10.3 | 16441 | 0.063 | 20.6 | 0.15 | 71.0 | 223 |
| 2006 | 3a | 14.8 | 68.5 | 52.9 | 12.5 | 3.1 | 17.0 | 7.5 | 7.3 | 2.2 | 1.3 | 128 | 0.59 | 2.41 | 10.16 | 0.92 | 0.10 | 0.31 | 14.6 | 15334 | 0.106 | 33.4 | 0.16 | 118.2 | 261 |
| 2006 | 3b | 8.9 | 76.1 | 58.9 | 13.9 | 3.3 | 14.9 | 7.5 | 7.2 | 1.4 | 0.8 | 99 | 0.33 | 1.42 | 8.08 | 1.10 | 113.04 | 0.26 | 10.4 | 16826 | 0.056 | 14.3 | 0.14 | 59.8 | 187 |
| 2006 | 4a | 13.0 | 75.0 | 51.9 | 12.8 | 10.3 | 12.0 | 7.2 | 7.0 | 1.5 | 0.9 | 94 | 0.45 | 2.67 | 9.57 | 0.62 | 0.17 | 0.13 | 8.3 | 15228 | 0.045 | 15.8 | 0.15 | 40.3 | 197 |
| 2006 | 4b | 12.0 | 72.1 | 55.0 | 17.0 | 0.2 | 15.9 | 6.5 | 6.1 | 1.4 | 0.8 | 103 | 0.49 | 2.10 | 7.77 | 0.70 | 0.12 | 0.11 | 9.7 | 13952 | 0.044 | 17.4 | 0.13 | 46.3 | 202 |
| 2007 | 5a | 13.0 | 72.6 | 47.8 | 11.3 | 13.6 | 14.4 | 7.3 | 6.3 | 1.7 | 1.0 | 88 | 0.17 | 2.04 | 11.36 | 27.1 | 3.9 | 0 | 14.3 | 19482 | 0.060 | 17.1 | 0.12 | 52.3 | 111 |
| 2007 | 5b | 12.9 | 70.2 | 44.5 | 12.4 | 13.2 | 17.0 | 7.6 | 6.5 | 1.4 | 0.8 | 75 | 0.12 | 1.66 | 10.67 | 29.10 | 2.10 | 0 | 13.4 | 20404 | 0.031 | 19.1 | 0.16 | 73.2 | 100 |
| 2007 | 6a | 12.0 | 70.7 | 48.5 | 10.5 | 11.7 | 17.3 | 7.4 | 6.8 | 1.9 | 1.1 | 113 | 0.23 | 2.63 | 9.73 | 29.10 | 2.50 | 0 | 12.2 | 15431 | 0.038 | 20.1 | 0.26 | 52.0 | 177 |
| 2007 | 6b | 17.0 | 66.2 | 44.3 | 14.9 | 7.0 | 16.8 | 7.6 | 6.9 | 1.5 | 0.9 | 87 | 0.24 | 1.73 | 10.34 | 22.50 | 1.60 | 0.07 | 8.1 | 10936 | 0.048 | 15.6 | 0.19 | 39.2 | 150 |

2008 data not available

**Table S3** Reproducibility evaluation based on five selected peaks

|  | MeOh/Water Fraction | | | | | | Chloroform Fraction | | | | |
| --- | --- | --- | --- | --- | --- | --- | --- | --- | --- | --- | --- |
| Peak No. | t_R(min)_ | *m/z* | Δm (mDa) | S.D.t_R_ | Peak area  % R.S.D | | t_R(min)_ | *m/z* | Δm  (mDa) | S.D.t_R_ | Peak area  % R.S.D |
| ESI+ | | | | | | | | | | | |
| 1 | 1.03 | 130.9 | 0.25 | 0.01 | | 4.1 | 6.68 | 316.2 | 2.46 | 0.02 | 8.8 |
| 2 | 4.52 | 166.0 | 0.15 | 0.04 | | 3.0 | 11.90 | 181.1 | 1.46 | 0.02 | 1.8 |
| 3 | 6.61 | 205.0 | 0.76 | 0.01 | | 8.8 | 13.57 | 209.2 | 1.48 | 0.01 | 5.2 |
| 4 | 12.01 | 579.1 | 2.85 | 0.01 | | 10.9 | 17.85 | 496.3 | 3.47 | 0.01 | 4.7 |
| 5 | 19.61 | 338.3 | 1.80 | 0.02 | | 2.4 | 18.80 | 391.3 | 3.27 | 0.02 | 1.2 |
| ESI- | | | | | | | | | | | |
| 1 | 2.26 | 115.1 | 0.10 | 0.06 | | 5.9 | 9.92 | 201.1 | 0.21 | 0.02 | 6.6 |
| 2 | 5.36 | 312.1 | 1.41 | 0.03 | | 5.2 | 12.82 | 169.1 | 0.31 | 0.01 | 8.2 |
| 3 | 10.89 | 187.1 | 0.44 | 0.03 | | 6.2 | 15.66 | 311.2 | 0.64 | 0.01 | 8.8 |
| 4 | 12.31 | 485.3 | 0.91 | 0.02 | | 12.7 | 17.52 | 564.3 | 1.81 | 0.04 | 3.2 |
| 5 | 14.59 | 265.2 | 0.3 | 0.02 | | 3.9 | 18.35 | 559.5 | 2.61 | 0.02 | 6.1 |
| ESI- acid | | | | | | | | | | | |
| 1 | 4.59 | 164.0 | 2.78 | 0.01 | | 2.4 | 11.08 | 221.1 | 0.50 | 0.03 | 8.0 |
| 2 | 5.27 | 218.1 | 1.40 | 0.02 | | 4.6 | 12.80 | 167.1 | 0.10 | 0.02 | 7.8 |
| 3 | 7.60 | 431.2 | 4.49 | 0.01 | | 4.6 | 16.53 | 311.2 | 0.35 | 0.01 | 5.3 |
| 4 | 11.03 | 541.1 | 0.70 | 0.02 | | 1.8 | 16.56 | 295.2 | 0.53 | 0.01 | 4.9 |
| 5 | 16.90 | 387.1 | 3.4 | 0.02 | | 8.2 | 18.39 | 559.5 | 0.91 | 0.02 | 8.1 |

S.D.t_R_: standard deviation of retention times in 14 QC samples; % R.S.D: relative standard deviation of peak areas in 14 QC samples; Δm (mDa): the mass errors for each peak in mDa; ESI: electrospray ionization.
